# Enhanced detection of metastatic prostate cancer cells in human plasma with lipid bodies staining

**DOI:** 10.1186/1471-2407-14-91

**Published:** 2014-02-15

**Authors:** Ranjana Mitra, Oscar B Goodman, Thuc T Le

**Affiliations:** 1 Roseman University of Health Sciences, 11 Sunset Way, Henderson, NV 89014, USA; 2 Desert Research Institute, 10530 Discovery Drive, Las Vegas, NV 89135, USA

**Keywords:** Cancer energy metabolism, Coherent anti-Stokes Raman microscopy, Circulating tumor cell, Flow cytometry, Lipid bodies, Prostate cancer, Protein lysine acetylation, Protein O-linked glycosylation, Proteomics

## Abstract

**Background:**

Reprogramming of energy metabolism of malignant cancer cells confers competitive advantage in growth environments with limited resources. However, not every process of cancer development is associated with competition for resources. During hematogenous transport, cancer cells are exposed to high levels of oxygen and nutrients. Does energy metabolism of cancer cells change as a function of exposure to the bloodstream? Could such changes be exploited to improve the detection of circulating tumor cells (CTC)? These questions have clinical significance, but have not yet been sufficiently examined.

**Methods:**

The energy metabolism was examined as a function of incubation in nutrient-rich plasma in prostate metastatic cancer cells LNCaP and non-transformed prostate epithelial cells RWPE1. Uptake kinetics of a fluorescent glucose analog (2-NBD) and lipophilic dyes (DiD & Bodipy) were measured in both cell lines, as well as in peripheral blood mononuclear cells (PBMC).

**Results:**

LNCaP cells exhibited hyper-acetylation of low molecular weight proteins compared to RWPE1 cells. Following plasma incubation, protein lysine acetylation profile was unchanged for LNCaP cells while significantly altered for RWPE1 cells. O-linked glycosylated protein profiles were different between LNCaP and RWPE1 cells and varied in both cell lines with plasma incubation. Maximal respiration or glycolytic capacities was unchanged in LNCaP cells and impaired in RWPE1 cells following plasma incubation. However, the uptake rates of 2-NBD and DiD were insufficient for discrimination of LNCaP, or RWPE1 cells from PBMC. On the other hand, both RWPE1 and LNCaP cells exhibited intracellular lipid bodies following plasma incubation; whereas, PBMC did not. The presence of lipid bodies in LNCaP cells permitted retention of Bodipy dye and allowed discrimination of LNCaP cells from PBMC with flow cytometry.

**Conclusions:**

Despite clear differences in energy metabolism, metastatic prostate cancer cells could not be efficiently distinguished from non-transformed prostate epithelial cells using fluorescent glucose or lipid uptake kinetics. However, metastatic prostate cancer cells in plasma could be clearly distinguished from blood nucleated cells due to the presence of intracellular lipid bodies. Fluorescent labeling of lipid bodies permitted a simple and sensitive means for high throughput detection of metastatic prostate cancer cells in human plasma.

## Background

Reprogramming of cellular energy metabolism is a distinctive hallmark of malignant transformation [1]. Many cancerous cells are reliant on glycolysis rather than mitochondrial respiration for energy metabolism even in the presence of oxygen [2]. This phenomenon is known as aerobic glycolysis or Warburg’s effect to honor the observation first described by biochemist Otto Warburg in the early half of the 20th century [3]. The precise cause of aerobic glycolysis is still under investigation. However, sustained aerobic glycolysis is associated with the activation of oncogenes or loss of tumor suppressors [4]. Cellular energy metabolism pathway is intrinsically and dynamically linked to nutrient-sensing and signaling pathways. Therefore, reprogramming of cellular energy metabolism during tumorigenesis is expected to be coupled with alteration in nutrient-sensing and signaling pathway [5].

In the presence of oxygen, normal cells prefer oxidative phosphorylation over glycolysis to maximize ATP production per glucose molecule [6]. Under hypoxic condition, normal cells undergo lactic acid fermentation, or anaerobic glycolysis, where glucose is converted to energy and lactate. Hypoxic regions of a tumor mass arise due to high rates of cell proliferation and insufficient blood supply [7]. Hypoxic adaptation is critical for the survival and growth of a tumor [8]. While aerobic glycolysis is an inefficient means to generate ATP, it facilitates the accumulation of biomass essential for cell proliferation [6]. Aerobic glycolysis confers competitive advantage for cancer cells in a growth environment with limited resources [8]. Hypoxic adaptation of cancer cells persists even in the condition of oxygen abundance [9].

However, not every process of cancer development is associated with competition for resources. A critical step of cancer metastasis is hematogenous transport, where CTC are exposed to high levels of oxygen and nutrients [10]. Cancer metastasis is the primary cause of cancer-specific mortality [11,12]. CTC are promising therapeutic targets for the prevention of cancer mortality because they are highly accessible to anti-cancer pharmaceutical compounds [13]. Enumeration of CTC is being pursued as a means to monitor cancer progression and response to therapy [14]. Yet it is not clear how metastatic cancer cells, which have re-programed cellular energy metabolism for adaptation to hypoxic condition, deal with an environment rich in nutrients and oxygen like the bloodstream [9].

In this study, we examine nutrient-sensitive protein post-translational modifications and bioenergetics of LNCaP and RWPE1 cells of human prostate origin as a function of incubation in nutrient-rich plasma. We also examine the ability to uptake lipid and glucose of these cell lines in plasma and compare them to PBMC. Using this *in vitro* model system, we aim to infer the behavior of prostate CTC to design effective means for prostate CTC detection.

## Methods

### Cell lines and materials

LNCaP and RWPE1 cell lines were obtained from ATCC. LNCaP cells were maintained in RPMI media and RWPE1 cells were grown in Keratinocyte media supplemented with bovine pituitary extract and epidermal growth factor (Invitrogen, Carlsbad, CA) in a humidified incubator with 5% CO_2_. All cell culture media were supplemented with 10% FBS. Human sodium citrate pooled plasma was purchased from Bioreclamation Inc. (Westbury, NY). The peripheral blood mononuclear cells (PBMC) were isolated from whole heparinized blood by Ficoll plaque gradient centrifugation; buffy coat was collected and washed with PBS to remove platelets.

### 1D Western blots

**Figure 1:**
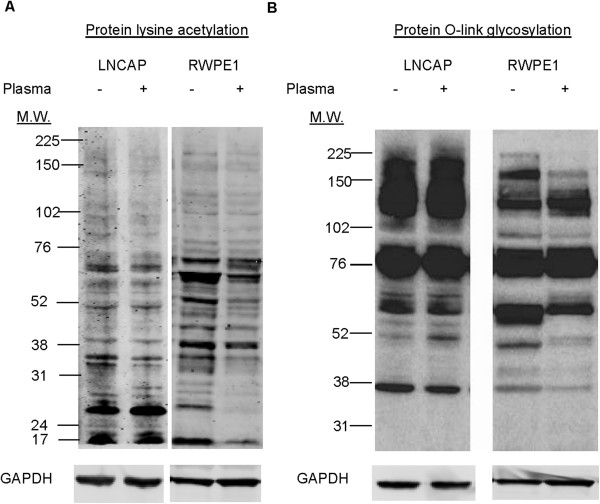
**1D Western blots of nutrient-sensitive protein post-translational modifications. (A)** Protein lysine acetylation profiles as a function of human plasma incubation of LNCaP and RWPE1 cells. **(B)** Protein O-linked glycosylation profiles as a function of human plasma incubation of LNCaP and RWPE1 cells. GAPDH serves as loading controls.

Total protein extracts were separated on 10% SDS-PAGE gels, transferred to nitrocellulose membranes, incubated first with primary antibodies against proteins of interest and then with secondary antibodies conjugated with HRP or labeled with Infrared Dye. Membranes were stripped, and re-incubated with antibodies against GAPDH for evaluation of loading controls. Primary antibodies were anti-acetylated lysine (1:1000, Cat. No. 9441S), GAPDH (1:10,000, Cat. No. 10R-G109A), and anti-O-linked N-acetylglucosamine (1:1000, Cat. No. 9875S) from Cell Signaling (Danvers, MA), Sigma Aldrich (Saint Louis, MO), Fitzgerald (Acton, MA) and Thermo Fisher Scientific (Lafayette, CO) respectively. Infrared fluorescently labeled secondary antibodies conjugated with IR dye 680 (Cat. No. 926–68070) and IR dye 800 (Cat. No. 926–32211) from LICOR Biosciences (Lincoln, NE) were used for detection using Odyssey CLx Imager. The experiments were repeated three times and one representative experiment is shown in Figure 1. In case of anti-O-linked N-acetylglucosamine HRP-linked IgM antibody was provided with the kit.

### 2D Western blots

2D Western blots were performed by Kendrick Laboratories (Madison, WI). The blots were wet in 100% methanol, rinsed briefly in Tween-20 Tris buffered saline (TTBS), and blocked for two hours in 5% bovine serum albumin (BSA) in TTBS. The blots were then incubated in primary antibodies including anti-acetylated lysine (Cat. No. 9441S) and anti-O-GlcNAc monoclonal antibody (Cat. No. 9875S) from Cell Signaling diluted 1:1000 in 2% BSA in TTBS overnight and rinsed 3 × 10 minutes in TTBS. The blots were then placed in secondary antibody (anti-mouse IgG HRP, GE, Cat. No. NA931V) diluted 1:2,000 in 2% BSA in TTBS for two hours, rinsed in TTBS as above, treated with ECL, and exposed to X-ray film. The spots to be sequenced were excised and sent to Applied Biomics (Hayward, CA) for protein identification with MALDI-TOF-MS.

### Bioenergetics of LNCaP and RWPE1

RWPE1 and LNCaP (30,000 to 40,000) cells were plated on the poly-D-Lysine (Sigma Aldrich-Saint Louis, MO) coated XL24 Seahorse bioanalyzer tissue culture 24 well plates. At 5 hours after plating, cells were incubated for 12 hours with 50% plasma or used as controls. Bioenergetics of LNCaP/RWPE1 was determined using the XF Cell Mito Stress Test Kit and a XF24-3 Analyzer (Seahorse Bioscience, North Billerica, MA) following published protocols [15]. Bioenergetics experiments were performed at the UCLA’s Cellular Bioenergetics Core Facilities. At least 48 repeated measurements were performed per experimental condition. The concentration of Oligomycin, Carbonyl cyanide 4-trifluoromethoxy phenylhydrazone (FCCP), Rotenone and Myxothiazol used was 0.5 μM, 0.75 μM, 0.75 μM and 0.75 μM for LNCaP and 0.75 μM, 0.25 μM, 0.75 μM and 0.75 μM for RWPE1 respectively.

### Measurement of glucose and lipid uptake

To measure glucose and lipid uptake, fluorescently labeled glucose analogue and lipophilic dyes were used, respectively. All dyes were purchased from Molecular Probe (Life Technologies, Grand Island, NY). For glucose uptake measurement, 2-NBD glucose (Cat. No. N13195) at 12.5 μg/ml was incubated for 0 to 30 minutes at room temperature, washed and analyzed on a C6 Accuri flow cytometer (BD Bioscience, San Jose, CA). For lipid uptake measurement, either DiD dye (Cat. No. V-22887) at 1.25 μM or Bodipy FL C16 dye (Cat. No. D3821) at 1 μg/ml was used. Cells were incubated at room temperature for 0 to 30 minutes, then washed and analyzed with flow cytometry.

### Immunofluorescence labeling

For flow cytometry, cells were first stained with FITC conjugated CD45 antibody (Cat. No. 555482, BD Bioscience), then fixed and permeabilized with Intrasure kit (Cat. No. 641776, BD Bioscience), and finally labeled with AlexaFluor647 conjugated pan-cytokeratin antibody (Cat. No. 4528, Cell Signaling, Danvers, MA). For fluorescent imaging, cytokeratin was visualized by staining first with unconjugated primary antibodies (Cat. No. 8018, Santa Cruz Biotechnology, Santa Cruz, CA), then with TRITC-conjugated secondary antibodies.

### Coherent anti-stokes Raman scattering (CARS) microscopy

Vibrational frequency used for lipid bodies imaging was fixed at 2851 cm^-1^ using a custom-built multimodal CARS microscope described previously [16]. CARS microscopy is a sensitive label-free method for visualization of lipid bodies [17,18]. CARS lasers were also used for simultaneous two-photon fluorescence imaging. Epi-reflected signals were collected using a three-channel detector. Bandpass filters for FITC, TRITC, and CARS were 510/42 nm, 579/34 nm, and 736/128 nm, respectively.

## Results and discussion

### Protein acetylation of LNCaP cells was insensitive to plasma incubation

Protein acetylation and glycosylation are nutrient-sensitive post-translational modifications important for the regulation of cellular energy metabolism [5]. Using 1D Western blots, protein lysine acetylation profiles of LNCaP cells were examined following incubation with 50% human plasma for 24 hours (Figure 1A). Surprisingly, there was no significant change to protein acetylation profiles between untreated and plasma treated LNCaP cells. Compared to RWPE1 cells, LNCaP cells exhibited hyper-acetylation of proteins with low molecular weight. In contrast, non-transformed prostate epithelial RWPE1 cells exhibited significant changes to the protein lysine acetylation profiles between untreated and plasma treated cells. Interestingly, plasma treated RWPE1 cells exhibited de-acetylation of low molecular weight proteins compared to untreated RWPE1 cells.

### Protein O-linked glycosylation of LNCaP cells was insensitive to plasma incubation

1D Western blots were employed to examine protein O-linked glycosylation of LNCaP cells following incubation with 50% human plasma for 24 hours (Figure 1B). With the exception of a slight increase in O-linked glycosylation of a protein band at 52 kD, there was no other significant change to protein O-linked glycosylation profiles between untreated and plasma treated LNCaP cells. In contrast, RWPE1 cells exhibited several significant changes to the protein O-linked glycosylation profiles between untreated and plasma treated cells. Most notable are the de-glycosylation of a protein band at approximately 60 kD and a protein band at 52 kD following incubation of RWPE1 cells in 50% human plasma.

### Identification of lysine acetylated proteins with proteomics

**Figure 2:**
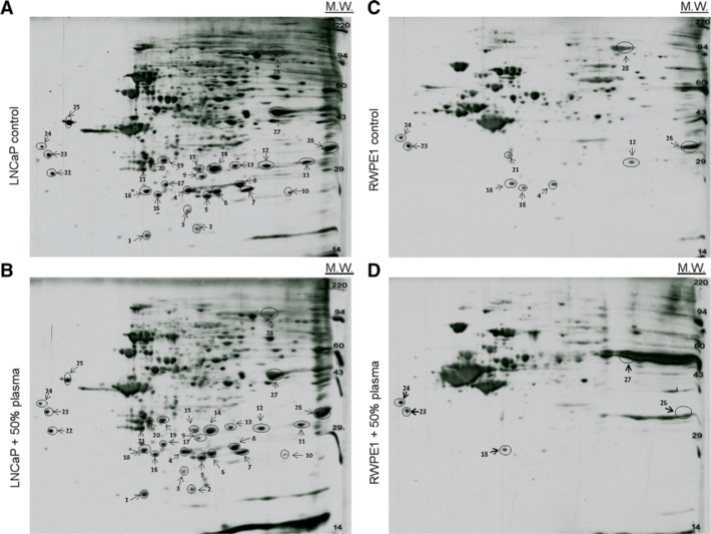
**2D Western blots of protein lysine acetylation profiles.** Protein lysine acetylation profiles of **(A)** untreated LNCaP cells, **(B)** LNCaP cells incubated with human plasma, **(C)** untreated RWPE1 cells, and **(D)** RWPE1 cells incubated with human plasma.

2D Western blots were employed for high resolution analysis of protein lysine acetylation profiles (Figure 2A-D). Compared to RWPE1 cells, LNCaP cells exhibited protein hyper-acetylation with significantly more immuno-positive spots for proteins with low molecular weight of 30 kD or less (Figure 2A, C). Plasma treatment did not significantly change the protein lysine acetylation profile of LNCaP cells (Figure 2A, B). In contrast, plasma treatment reduced lysine acetylation of four low molecular weight protein spots of RWPE1 cells (Figure 2C, D). Selective 2D gel spots matching the positions of lysine acetylated proteins were excised and used for protein identification with matrix-assisted laser desorption/ionization time-of-flight mass spectrometry (MALDI-TOF-MS). Identified acetylated proteins and their biological functions are listed in Table 1 and Additional file 1: Table S1. Expectedly, a number of acetylated proteins participate in energy metabolism pathway including mitochondrial fatty acid β-oxidation and glycolysis. Other acetylated proteins participate in antioxidation, stress response, cytoskeletal structures, and other biological functions. Interestingly, four low molecular weight protein spots that got de-acetylated following plasma incubation in RWPE1 cells were identified as enoyl-CoA hydratase (mitochondrial fatty acid β-oxidation), mitochondrial carrier homolog 2 (transport/apoptosis), actin (cytoskeletal structure), and peroxiredoxin-4 (antioxidation). Many proteins identified did not migrate with predicted molecular weights or isoelectric points due to possible protein post-translational modifications or degradation.

**Table 1:** Protein lysine acetylation as a function of plasma incubation

**Spot number**	**Protein name**	**LNCaP control**	**LNCaP + plasma**	**RWPE1 control**	**RWPE1 + plasma**	**Biological function**
1	Peroxiredoxin-2	√	√	-	-	Antioxidation
2	Protein DJ-1	√	√	-	-	Stress Response
3	Thioredoxin-dependent peroxide reductase, mitochondrial	√	√	-	-	Antioxidation
4	Enoyl-CoA hydratase, mitochondrial	√	√	√	-	Fatty Acid β-oxidation
5	Peroxiredoxin-6	√	√	-	-	Antioxidation
6	Triosephosphate isomerase	√	√	-	-	Glycolysis
7	Triosephosphate isomerase	√	√	-	-	Glycolysis
8	Phosphoglycerate mutase 1	√	√	-	-	Glycolysis
9	78 kDa glucose-regulated protein	√	√	-	-	Unfolded Protein Response
10	40S ribosomal protein S8	√	√	-	-	Protein Synthesis
11	Prohibitin-2	√	√	-	-	Transcription Regulation
12	Mitochondrial carrier homolog 2	√	√	√	-	Mitochondrial Transport
13	S-formylglutathione hydrolase	√	√	-	-	Formaldehyde Catabolism
14	Delta (3,5)-Delta (2,4)-dienoyl-CoA isomerase, mitochondrial	√	√	-	-	Fatty Acid β-oxidation
15	26S proteasome non-ATPase regulatory subunit 14	√	√	-	-	26S Proteosome Assembly
16	Peroxiredoxin-4	√	√	√	-	Antioxidation
17	Proteasome activator complex subunit 1	√	√	-	-	Cell Differentiation
18	Prohibitin	√	√	√	√	DNA Synthesis
19	L-lactate dehydrogenase B chain	√	√	-	-	Glycolysis
20	Inorganic pyrophosphatase	√	√	-	-	Diphosphate Metabolism
21	Actin, cytoplasmic 1	√	√	√	-	Cytoskeletal Structure
22	Tropomyosin alpha-3 chain	√	√	-	-	Cytoskeletal Structure
23	40S ribosomal protein SA	√	√	√	√	40S Ribosome Assembly
24	Tropomyosin beta chain	√	√	√	√	Cytoskeletal Structure
25	78 kDa glucose-regulated protein	√	√	-	-	Unfolded Protein Response
26	Glyceraldehyde-3-phosphate dehydrogenase	√	√	√	√	Glycolysis
27	Alpha enolase	√	√	-	√	Glycolysis
28	Calnexin	√	√	√	-	Calcium Binding

### Identification of O-linked glycosylated proteins with proteomics

**Figure 3:**
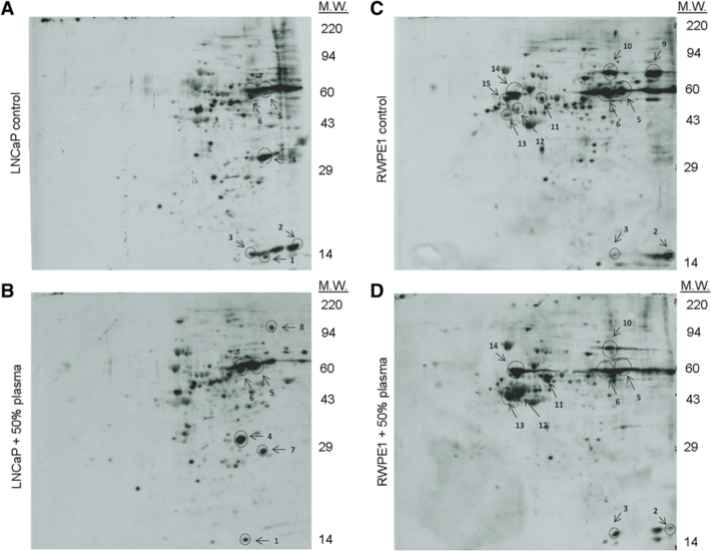
**2D Western blots of protein O-linked glycosylation profiles.** Protein O-linked glycosylation profiles of **(A)** untreated LNCaP cells, **(B)** LNCaP cells incubated with human plasma, **(C)** untreated RWPE1 cells, and **(D)** RWPE1 cells incubated with human plasma.

2D Western blots were employed for high resolution analysis of protein O-linked glycosylation profiles (Figure 3A-D). Plasma incubation induced significant changes to the protein O-linked glycosylation profiles of LNCaP cells. Most notably are the de-glycosylation of protein spots 2 and 3 of ~14 kD and glycosylation of protein spot 7 of ~29 kD (Figure 3A, B). Plasma incubation also induced significant changes to the protein O-linked glycosylation profiles of RWPE1 cells. Most notably are the de-glycosylation of protein spot 9 of ~80 kD and the reduced glycosylation of protein spots 5 and 6 of ~60 kD (Figure 3C, D). In general, RWPE1 cells exhibited more O-linked glycosylated protein spots than LNCaP cells. Selective 2D gel spots matching the positions of O-linked glycosylated proteins were excised and used for protein identification with MALDI-TOF-MS. Identified O-linked glycosylated proteins and their biological functions are listed in Table 2 and Additional file 1: Table S2. For LNCaP cells, protein spots 2 and 3 were identified as histone 2B type 1-M, a component of the nucleosome. Protein spot 7 was identified as phosphoglycerate mutase 1, an enzyme of the glycolytic pathway. On the other hand, protein spot 9 of RWPE1 cells was identified as mitochondrial trifunctional enzyme subunit alpha, which is a critical enzyme for fatty acid β-oxidation pathway. Protein spots 5 and 6 were identified as keratin, type II cytoskeletal 6A and 6B, respectively. Proteomics data using 2D Western blots couldn’t identify any specific protein spot at 52 kD, whose O-linked glycosylation changed as a function of plasma incubation in 1D Western blot. It is likely that the glycosylation profile of the 1D gel band at 52 kD was the collective profile of multiple proteins with the same molecular weight.

**Table 2:** Protein O-linked glycosylation as a function of plasma incubation

**Spot number**	**Protein name**	**LNCaP control**	**LNCaP + plasma**	**RWPE1 control**	**RWPE1 + plasma**	**Biological function**
1	UPF0556 protein C19orf10	√	√	-	-	Unfolded Protein Response
2	Histone H2B type 1-M	√	-	√	√	Nucleosome Assembly
3	Histone H2B type 1-M	√	-	√	√	Nucleosome Assembly
4	Delta (3,5)-Delta (2,4)-dienoyl-CoA isomerase, mitochondrial	√	√	-	-	Fatty Acid Metabolim
5	Keratin, type II cytoskeletal 6A	√	√	√	√	Cytoskeletal Structure
6	Keratin, type II cytoskeletal 6B	√	√	√	√	Cytoskeletal Structure
7	Phosphoglycerate mutase 1	-	√	-	-	Glycolysis
8	Elongation factor 2	-	√	-	-	Protein Synthesis
9	Trifunctional enzyme subunit alpha, mitochondrial	-	-	√	-	Fatty Acid β-oxidation
10	Procollagen galactosyltransferase 1	-	-	√	√	ECM Organization
11	Keratin, type II cytoskeletal 7	-	-	√	√	Cytoskeletal Structure
12	Keratin, type I cytoskeletal 14	-	-	√	√	Cytoskeletal Structure
13	Keratin, type I cytoskeletal 17	-	-	√	√	Cytoskeletal Structure
14	Vimentin	-	-	√	√	Cytoskeletal Structure
15	Vimentin	-	-	√	-	Cytoskeletal Structure

### Maximal respiration and glycolytic capacities of LNCaP cells were insensitive to plasma incubation

**Figure 4:**
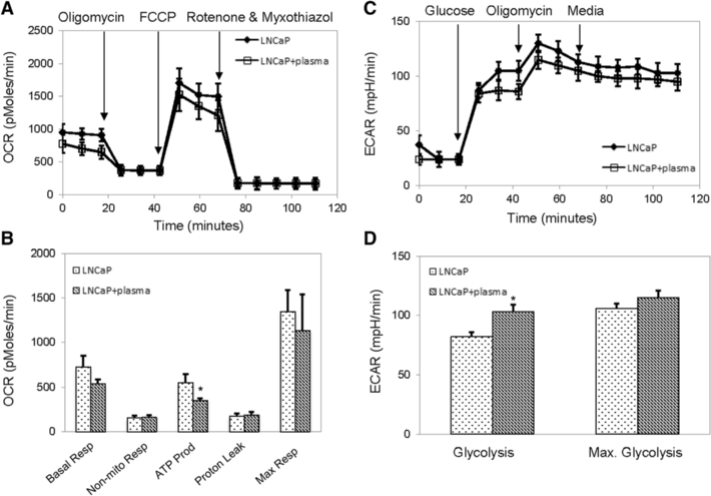
**Bioenergetics of LNCaP cells as a function of plasma incubation. (A)** Real-time oxygen consumption rates (OCR) and **(B)** average values of key parameters for the evaluation of mitochondrial functions of LNCaP cells. **(C)** Real-time extracellular acidification rates (ECAR) and **(D)** average values of key parameters for the evaluation of glycolytic function of LNCaP cells. Error bars are standard deviation across 48 repeats per experimental condition. Asterisk indicates P-value < 0.05 determined with paired Student’s *t*-test against untreated control.

**Figure 5:**
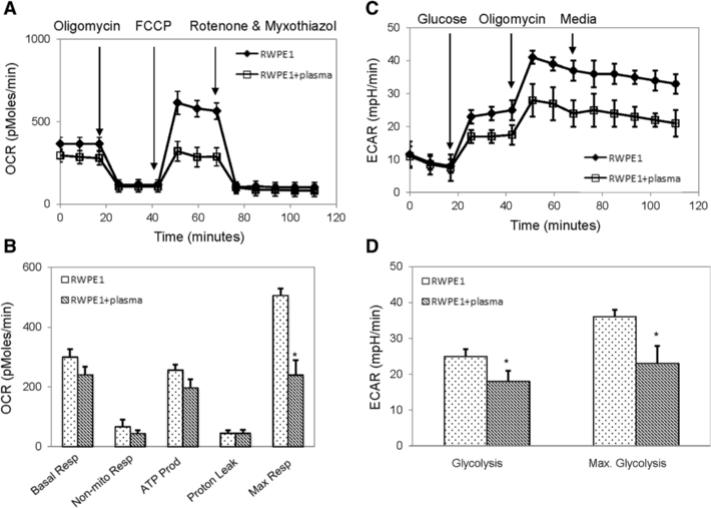
**Bioenergetics of RWPE1 cells as a function of plasma incubation. (A)** Real-time oxygen consumption rates (OCR) and **(B)** average values of key parameters for the evaluation of mitochondrial functions of RWPE1 cells. **(C)** Real-time extracellular acidification rates (ECAR) and **(D)** average values of key parameters for the evaluation of glycolytic function of RWPE1 cells. Error bars are standard deviation across 48 repeats per experimental condition. Asterisk indicates P-value < 0.05 determined with paired Student’s *t*-test against untreated control.

Bioenergetics of LNCaP and RWPE1 cells were evaluated as a function of plasma incubation. Using an extracellular flux analyzer, mitochondrial function was evaluated by measuring in real time the oxygen consumption rates (OCR) and glycolytic function was evaluated by measuring in real time the extracellular acidification rates (ECAR) (Additional file 1: Figure S1A, B). Incubation with plasma slightly reduced ATP production of LNCaP cells while having no statistically significant effect on their maximal mitochondrial respiration capacity (Figure 4A, B). Incubation with plasma also slightly increased glycolysis of LNCaP cells while having no statistically significant effect on their maximal glycolytic capacity (Figure 4C, D). In contrast, incubation with plasma slightly reduced ATP production of RWPE1 cells while severely reduced their maximal mitochondrial respiration capacity by over 50% (p-value <0.05) (Figure 5A, B). Incubation with plasma also reduced both the average glycolysis rate and maximal glycolytic capacity of RWPE1 cells by over 30% (p-value <0.05) (Figure 5C, D).

### Plasma incubation repressed glucose uptake and enhanced lipid uptake of LNCaP cells

**Figure 6:**
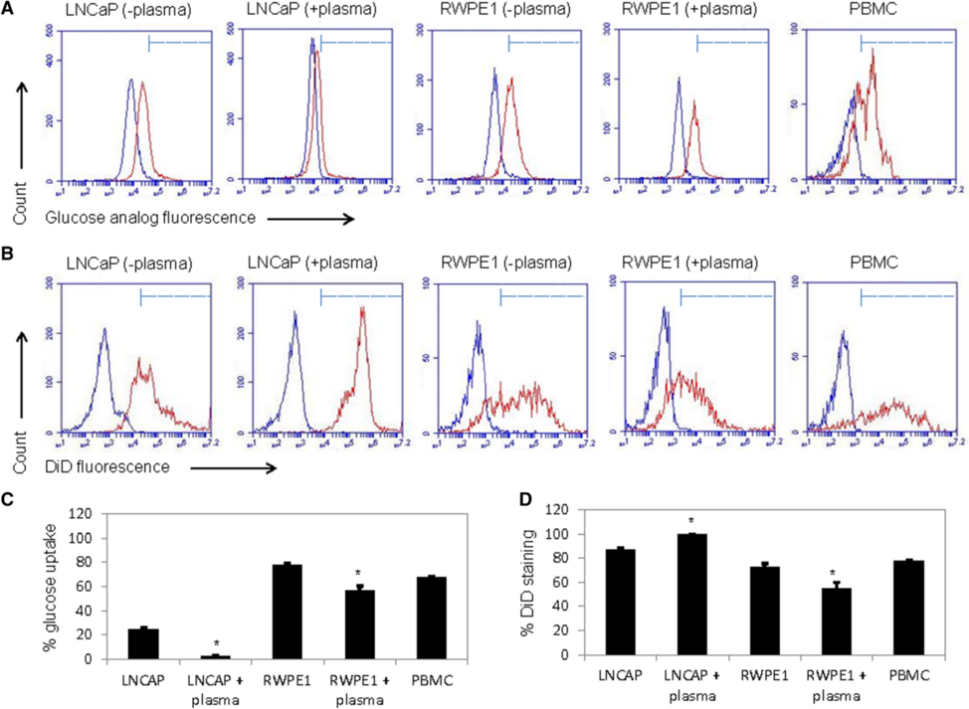
**Comparison of glucose and lipid uptake between RWPE1, LNCaP and PBMC. (A)** Glucose and **(B)** lipid uptake of LNCaP, RWPE1 and PBMC cells. Blue lines represent control cells without incubation with neither fluorescent glucose analog nor DiD. Red lines represent cells incubated with either fluorescent glucose analog **(A)** or DiD **(B)**. Dashed lines with bar heads indicate the cut-off fluorescence intensity values that separate stained cells from unstained cells. Cut-off values were chosen to exclude >95% of control unstained cells. Cells were incubated with plasma for 16 hours, then with fluorescent glucose analog or with lipophilic DiD dye for 15 minutes. Data presented are of one set of flow cytometry experiment. **(C)** and **(D)** are graphical presentation of average flow cytometry data of at least 4 repeats per experimental condition for glucose and lipid uptake, respectively. Error bars are standard deviation across 4 repeated measurements. Asterisk indicates P-value < 0.05 determined with paired Student’s *t*-test against untreated control.

Next, glucose and lipid uptake of LNCaP cells were evaluated using a fluorescent glucose analog and a lipid stain dye (DiD) and conventional flow cytometry. The difference between glucose and lipid uptake of LNCaP cells, RWPE-1 cells, and peripheral blood mononuclear cells (PBMC) was also evaluated to determine whether such difference could be exploited for their detection (Figure 6A, B). We found that plasma incubation repressed uptake of fluorescent glucose analog of more than 90% in LNCaP cells and less than 30% of RWPE1 cells (Figure 6C, Additional file 1: Figure S2, Additional file 1: Figure S3). Nearly 70% of PBMC exhibited uptake of the fluorescent glucose analog. In contrast, plasma incubation enhanced DiD staining of LNCaP cells by 14% (86% staining without plasma incubation versus 100% staining with plasma incubation) (Figure 6D, Additional file 1: Figure S4, Additional file 1: Figure S5). On the other hand, plasma incubation repressed DiD staining of RWPE1 cells by approximately 17%. Nearly 80% of PBMC stained positively with DiD **(**Figure 6B, Additional file 1: Figure S6). Our data revealed that plasma incubation repressed glucose uptake of LNCaP cells while enhanced their lipid uptake.

### Bodipy staining of LNCaP cells was preserved after fixation and permeabilization

**Figure 7:**
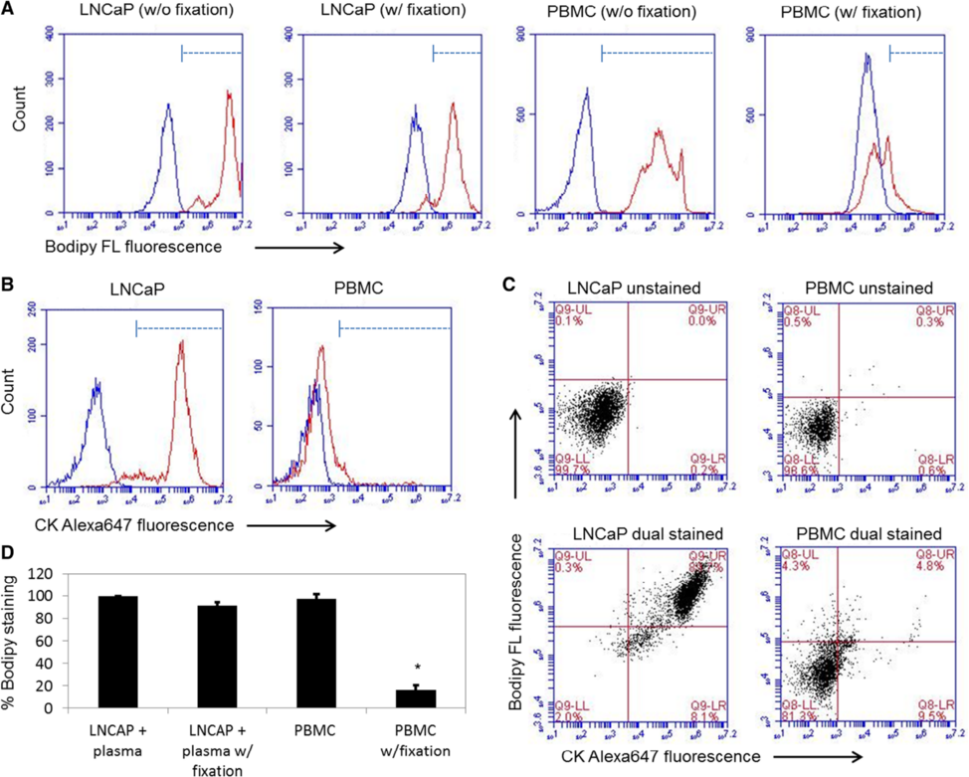
**Effect of fixation on lipophilic dye retention in LNCaP and PBMC cells. (A)** Both LNCaP and PBMC cells were labeled with Bodipy for 5 min, after labeling half of the cells were fixed and permeabilized using intrasure kit. **(B)** Permeabilized cells were labeled with pan-cytokeratin antibody tagged with Alexa647 for 30 min at room temperature. **(B)** Blue lines represent LNCaP or PBMC cells without staining with Bodipy FL or immunolabeled with pan-cytokeratin antibody tagged with Alexa 647. Red lines represent LNCaP or PBMC cells stained with Bodipy FL or immunolabeled with pan-cytokeratin antibody tagged with Alexa 647. **(C)** Two-color flow cytometry data for dual-stained LNCaP and PBMC cells. Data presented are of one set of flow cytometry experiments. **(D)** The average values of four experiments are shown in a graphical format. Asterisk indicates P-value < 0.05 determined with paired Student’s *t*-test against untreated control.

To improve lipid staining, DiD was replaced with Bodipy, a fluorescent fatty acid analog that undergoes native like transport and metabolism. Using flow cytometry, we found that both LNCaP cells (incubated over night with plasma) and PBMC achieved nearly 100% staining with Bodipy after 5 minutes of staining (Figure 7A). However, after fixation and permeabilization, Bodipy staining was retained for more than 90% of LNCaP cells compared to less than 20% of PBMC. A second stain with cytokeratin conjugated to Alexa Fluor 647 confirmed that up to 90% of LNCaP cells and only 5% of PBMC were stained positively for both Bodipy and cytokeratin following fixation and permeabilization protocol (Figure 7B, C). Clearly, while both LNCaP cells and PBMC exhibited strong affinity for Bodipy, only cytokeratin-positive LNCaP cells were able to significantly retain Bodipy after fixation and permeabilization.

### Formation of lipid bodies in LNCaP cells following plasma incubation

**Figure 8:**
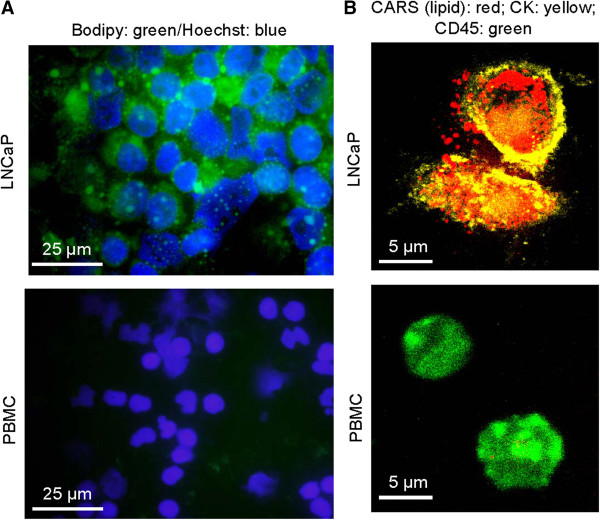
**Visualization of lipid bodies in LNCaP cells. (A)** Widefield fluorescent images of LNCaP and PBMC cells stained with Bodipy (green) for lipid bodies and Hoechst (blue) for nuclei. **(B)** CARS (red) images of lipid bodies and two-photon fluorescence (yellow, green) images of immunofluorescently labeled LNCaP cells (yellow) and PBMC cells (green). LNCaP cells were identified with TRITC conjugated primary antibodies against cytokeratin (CK). PBMC cells were identified with FITC conjugated primary antibodies against CD45.

Widefield fluorescent microscopy confirmed the ability to retain Bodipy staining after fixation and permeabilization of LNCaP cells and lack-there-of in PBMC (Figure 8A). Interestingly, Bodipy staining revealed granulated structures in the cytoplasm of LNCaP cells. Using coherent anti-Stokes Raman scattering (CARS) microscopy, a label-free imaging technique for lipid visualization [17], and two-photon fluorescent (TPF) microscopy, we observed the present of lipid bodies in LNCaP cells but not in PBMC (Figure 8B). Taken together, our data revealed that Bodipy was taken up by both LNCaP cells and PBMC along with plasma lipids. However, only in LNCaP cells did Bodipy and plasma lipids formed into lipid bodies. These lipid bodies remained within LNCaP cells even after cell fixation and permeabilization. In contrast, Bodipy and plasma lipids did not form into lipid bodies in PBMC, thus, escaped PBMC following cells fixation and permeabilization (Figure 8B & Figure 7D). The difference in the ability to form lipid bodies between LNCaP cells and PBMC permits clear differentiation of these cell types through the use of a fluorescent fatty acid analog Bodipy.

### Formation of lipid bodies in both RWPE1 and LNCaP cells following plasma incubation

Both RWPE1 and LNCaP cells accumulated intracellular lipid bodies after incubation with plasma (Additional file 1: Figure S7) [19]. The presence of lipid bodies caused lipophilic stains, such as Bodipy (data not shown) and Oil Red O, to remain even after cell fixation in both RWPE1 and LNCaP cells. Therefore, the use of lipid staining together with cell fixation and permeabilization did not permit the differentiation of RWPE1 from LNCaP cells.

## Conclusions

In this study, we reported that nutrient-sensing mechanism and bioenergetics of metastatic prostate LNCaP cells were insensitive to plasma incubation. Compared to non-transformed prostate epithelial RWPE1 cells, LNCaP cells exhibited hyper-acetylation of proteins with low molecular weights. These proteins participate in vital functions including mitochondrial fatty acid β-oxidation, transport, cytoskeletal structure, and antioxidation. Plasma incubation did not affect protein lysine acetylation profiles of LNCaP cells while significantly altered those of RWPE1 cells. On the other hand, LNCaP cells exhibited reduced O-link glycosylation of many cytoskeletal proteins compared to RWPE1. Plasma incubation reduced O-link glycosylation of histone H2B proteins while increased O-linked glycosylation of a glycolytic protein phosphoglycerate mutase 1 in LNCaP cells. In contrast, plasma incubation of RWPE1 cells exhibited reduced O-link glycosylation of cytoskeletal proteins keratin type 2 and a mitochondrial trifunctional enzyme, a protein critical for fatty acid β-oxidation. Plasma incubation did not affect maximal respiration and glycolytic capacities of LNCaP cells while significantly reduced these capacities of RWPE1 cells. In summary, bioenergetics and protein lysine acetylation profiles of LNCaP cells were less sensitive to plasma incubation compared to RWPE1 cells.

We also evaluated whether the difference in nutrient sensing and bioenergetics between LNCaP cells and RWPE 1 cells could affect their uptake kinetics of glucose and lipid. Following plasma incubation, uptake kinetics of a fluorescent glucose analog was significantly diminished in LNCaP cells but not in RWPE1 cells. In contrast, fluorescent lipid staining improved for LNCaP cells and declined for RWPE1 cells following plasma incubation. Nonetheless, the difference in glucose and lipid uptake kinetics between LNCaP and RWPE1 cells, with and without plasma incubation, were insufficient to be used for the discrimination of one cell type against the other. Our data concurred with an independent study where the uptake rates of glucose and palmitate were found to be comparable for malignant and benign prostate cell lines [20]. Thus, the affinity for glucose and lipids of LNCaP cells and RWPE1 cells did not mirror their clear difference in nutrient sensing and bioenergetics.

Increased affinity for lipid by LNCaP cells in plasma presents a unique opportunity for its detection. Interestingly, we observed that cell fixation and permeabilization following lipid staining permitted differentiation of LNCaP cells from PBMC. Both LNCaP cells and PBMC exhibited high lipid uptake rates as shown in this study and the literature [20-22]. However, cell fixation and permeabilization significantly reduced lipid staining of PBMC while only slightly reduced lipid staining of LNCaP cells. Consequently, LNCaP cells exhibited significantly higher lipid staining than PBMC. A key contributor to this observation was the presence of lipid bodies in LNCaP cells and lack-there-of in PMBC, which permitted retention of lipid stain following cell fixation and permeabilization. Compared with cytokeratin, a marker for prostate and epithelial cells, Bodipy lipid stain was equally effective in differentiating LNCaP cells from PBMC using both flow cytometry and fluorescence microscopy.

Lipid staining provides a simple means for high throughput detection of cancer cells in plasma. Previously, we reported label-free detection with CARS microscopy of lipid-rich prostate CTC in the peripheral blood samples of metastatic prostate cancer patients [19]. Accumulation of lipid bodies in cancer cells exposed to blood plasma appears to be a conserved property for various type of cancer cells of epithelial origins [19,23]. CARS microscopy is ideal for the detection of lipid-rich cells [24]. In recent years, CARS microscopy has been increasingly employed for imaging of primary tumors [25-30]. However, CARS microscopy is currently not yet available for clinical applications due to its large footprint, operation complexity, and high cost [18]. In contrast, flow cytometry and fluorescent microscopy are now available in most clinical pathology laboratories. Simple lipid staining of peripheral blood samples of cancer patients could provide a rapid means for initial screening for the presence of CTC. Future multicolor combinatorial staining of various markers including lipid, cytokeratin, CD45, epithelial cell adhesion molecule, could improve sensitivity and accuracy for CTC detection using flow cytometry and microscope-based cytometry.

## Abbreviations

CARS: Coherent anti-Stokes Raman scattering; CTC: Circulation tumor cells; MALDI-TOF-MS: Matrix-assisted laser desorption/ionization time-of-flight mass spectrometry.

## Competing interests

The authors (OBG & TTL) hold a US patent (US2013/0078667A1) on the methods to detect and isolate CTC.

## Authors’ contributions

RM and TTL designed experiments. OBG and TTL contributed reagents, samples, and analytical tools. RM and TTL performed experiments and analyzed data. RM and TTL prepared the manuscript. All authors read and approved final manuscript.

## Pre-publication history

The pre-publication history for this paper can be accessed here:

http://www.biomedcentral.com/1471-2407/14/91/prepub

## Supplementary Material

Additional file 1: Table S1 Lysine acetylated proteins identified with MALDI-TOF-MS. **Table S2.** O-linked glycosylated proteins identified with MALDI-TOF-MS. **Figure S1**. Graphical representation of measurements for energy metabolism. (A) Schematic depiction of how basal and maximum respiration values are calculated (B) schematic display to show the calculation of glycolysis and maximum glycolysis. **Figure S2.** Glucose uptake kinetics of LNCAP cells. Time course of (0-30 min) of glucose analog uptake kinetics is shown with and without plasma incubation. Data presented are from one single flow cytometry measurement per experimental condition. **Figure S3.** Glucose uptake kinetics of RWPE-1 cells. Time course of (0-30 min) of glucose analog uptake kinetics is shown with and without plasma incubation. Data presented are from one single flow cytometry measurement per experimental condition. **Figure S4.** Lipid uptake kinetics of LNCAP cells. Time course of (0-30min) of DiD uptake kinetics is shown with and without plasma incubation. Data presented are from one single flow cytometry measurement per experimental condition. **Figure S5.** Lipid uptake kinetics of RWPE-1 cells. Time course of (0-30 min) of DiD uptake kinetics is shown with and without plasma incubation. Data presented are from one single flow cytometry measurement per experimental condition. **Figure S6.** Uptake kinetics of PBMCs for glucose (A) and lipids (B). Time courses of (0-30 min) for fluorescent glucose analog and lipophilic DiD dye uptake kinetics are shown. Data presented are from one single flow cytometry measurement per experimental condition. **Figure S7.** Lipid droplet accumulation in RWPE1 and LNCaP cells following plasma incubation. RWPE1 and LNCaP cells were incubated for 12 hours with 50% plasma, then fixed, permeabilized and stained with Oil Red O. Click here for file
