# Supplementary material for: Enhanced detection of metastatic prostate cancer cells in human plasma with lipid bodies staining
Source: BMC Cancer. 2014 Feb 15;14:91. doi: 10.1186/1471-2407-14-91 (PMC3931481; doi:10.1186/1471-2407-14-91)
Supplement: Additional file 1: Table S1 — Lysine acetylated proteins identified with MALDI-TOF-MS. Table S2. O-linked glycosylated proteins identified with MALDI-TOF-MS. Figure S1. Graphical representation of measurements for energy metabolism. (A) Schematic depiction of how basal and maximum respiration values are calculated (B) schematic display to show the calculation of glycolysis and maximum glycolysis. Figure S2. Glucose uptake kinetics of LNCAP cells. Time course of (0-30 min) of glucose analog uptake kinetics is shown with and without plasma incubation. Data presented are from one single flow cytometry measurement per experimental condition. Figure S3. Glucose uptake kinetics of RWPE-1 cells. Time course of (0-30 min) of glucose analog uptake kinetics is shown with and without plasma incubation. Data presented are from one single flow cytometry measurement per experimental condition. Figure S4. Lipid uptake kinetics of LNCAP cells. Time course of (0-30min) of DiD uptake kinetics is shown with and without plasma incubation. Data presented are from one single flow cytometry measurement per experimental condition. Figure S5. Lipid uptake kinetics of RWPE-1 cells. Time course of (0-30 min) of DiD uptake kinetics is shown with and without plasma incubation. Data presented are from one single flow cytometry measurement per experimental condition. Figure S6. Uptake kinetics of PBMCs for glucose (A) and lipids (B). Time courses of (0-30 min) for fluorescent glucose analog and lipophilic DiD dye uptake kinetics are shown. Data presented are from one single flow cytometry measurement per experimental condition. Figure S7. Lipid droplet accumulation in RWPE1 and LNCaP cells following plasma incubation. RWPE1 and LNCaP cells were incubated for 12 hours with 50% plasma, then fixed, permeabilized and stained with Oil Red O. [file 1471-2407-14-91-S1.docx]

Supplementary data

for

**Enhanced detection of metastatic prostate cancer cells in human plasma with lipid bodies staining**

Ranjana Mitra^1^, Oscar B. Goodman^1*^, Thuc T. Le^2*^

^1^Roseman University of Health Sciences, 11 Sunset Way, Henderson, NV 89014, USA

^2^Desert Research Institute, 10530 Discovery Drive, Las Vegas, NV 89135, USA.

*To whom correspondence should be addressed: [ogoodman@roseman.edu](mailto:ogoodman@roseman.edu) & [thuc@uchicago.edu](mailto:thuc@uchicago.edu)

**Table S1. Lysine acetylated proteins identified with MALDI-TOF-MS**

| **Spot number** | **Protein Name** | **Accession No.** | **Protein MW** | **Protein PI** | **Peptide Count** | **Protein Score** | **Protein Score C.I.%** | **Total Ion Score** | **Total Ion C.I.%** |
| --- | --- | --- | --- | --- | --- | --- | --- | --- | --- |
| 1 | Peroxiredoxin-2 | PRDX2 | 21,878 | 5.7 | 10 | 690 | 100 | 614 | 100 |
| 2 | Protein DJ-1 | PARK7 | 19,879 | 6.3 | 9 | 352 | 100 | 289 | 100 |
| 3 | Thioredoxin-dependent peroxide reductase, mitochondrial | PRDX3 | 27,675 | 7.7 | 8 | 392 | 100 | 346 | 100 |
| 4 | Enoyl-CoA hydratase, mitochondrial | ECHM | 31,367 | 8.3 | 10 | 404 | 100 | 347 | 100 |
| 5 | Peroxiredoxin-6 | PRDX6 | 25,019 | 6.0 | 14 | 621 | 100 | 505 | 100 |
| 6 | Triosephosphate isomerase | TPIS | 30,772 | 5.7 | 13 | 392 | 100 | 301 | 100 |
| 7 | Triosephosphate isomerase | TPIS | 30,772 | 5.7 | 14 | 653 | 100 | 549 | 100 |
| 8 | Phosphoglycerate mutase 1 | PGAM1 | 28,786 | 6.7 | 12 | 746 | 100 | 652 | 100 |
| 9 | 78 kDa glucose-regulated protein | GRP78 | 72,288 | 5.1 | 14 | 1020 | 100 | 950 | 100 |
| 10 | 40S ribosomal protein S8 | RS8 | 24,190 | 10.3 | 6 | 363 | 100 | 340 | 100 |
| 11 | Prohibitin-2 | PHB2 | 33,276 | 9.8 | 12 | 505 | 100 | 425 | 100 |
| 12 | Mitochondrial carrier homolog 2 | MTCH2 | 33,309 | 8.3 | 10 | 298 | 100 | 238 | 100 |
| 13 | S-formylglutathione hydrolase | ESTD | 31,443 | 6.5 | 8 | 695 | 100 | 651 | 100 |
| 14 | Delta(3,5)-Delta(2,4)-dienoyl-CoA isomerase, mitochondrial | ECH1 | 35,793 | 8.2 | 15 | 544 | 100 | 437 | 100 |
| 15 | 26S proteasome non-ATPase regulatory subunit 14 | PSDE | 34,555 | 6.1 | 7 | 403 | 100 | 372 | 100 |
| 16 | Peroxiredoxin-4 | PRDX4 | 30,521 | 5.9 | 9 | 455 | 100 | 403 | 100 |
| 17 | Proteasome activator complex subunit 1 | PSME1 | 28,705 | 5.8 | 17 | 569 | 100 | 446 | 100 |
| 18 | Prohibitin | PHB | 29,786 | 5.6 | 14 | 1150 | 100 | 1046 | 100 |
| 19 | L-lactate dehydrogenase B chain | LDHB | 36,615 | 5.7 | 14 | 685 | 100 | 583 | 100 |
| 20 | Inorganic pyrophosphatase | IPYR | 32,639 | 5.5 | 14 | 918 | 100 | 821 | 100 |
| 21 | Actin, cytoplasmic 1 | ACTB | 41,710 | 5.3 | 10 | 543 | 100 | 493 | 100 |
| 22 | Tropomyosin alpha-3 chain | TPM3 | 32,799 | 4.7 | 13 | 463 | 100 | 398 | 100 |
| 23 | 40S ribosomal protein SA | RSSA | 32,833 | 4.8 | 9 | 460 | 100 | 408 | 100 |
| 24 | Tropomyosin beta chain | TPM2 | 32,831 | 4.7 | 18 | 587 | 100 | 477 | 100 |
| 25 | 78 kDa glucose-regulated protein | GRP78 | 72,288 | 5.1 | 22 | 1140 | 100 | 999 | 100 |
| 26 | Glyceraldehyde-3-phosphate dehydrogenase | G3P | 36,030 | 8.6 | 10 | 793 | 100 | 733 | 100 |
| 27 | Alpha-enolase | ENOA | 47,139 | 7.0 | 11 | 434 | 100 | 378 | 100 |
| 28 | Calnexin | CALX | 67,526 | 4.5 | 5 | 231 | 100 | 208 | 100 |

Table legend: MW: molecular weight; PI: isoelectric point; C.I.: Confident interval

**Table S2. O-linked glycosylated proteins identified with MALDI-TOF-MS**

| **Spot number** | **Protein Name** | **Accession No.** | **Protein MW** | **Protein PI** | **Peptide Count** | **Protein Score** | **Protein Score C.I.%** | **Total Ion Score** | **Total Ion C.I.%** |
| --- | --- | --- | --- | --- | --- | --- | --- | --- | --- |
| 1 | Delta(3,5)-Delta(2,4)-dienoyl-CoA isomerase, mito | ECH1 | 35,793 | 8.2 | 16 | 520 | 100 | 402 | 100 |
| 2 | Phosphoglycerate mutase 1 | PGAM1 | 28,786 | 6.7 | 15 | 689 | 100 | 559 | 100 |
| 3 | Histone H2B type 1-M | H2B1M | 13,981 | 10.3 | 8 | 354 | 100 | 304 | 100 |
| 4 | UPF0556 protein C19orf10 | CS010 | 18,783 | 6.2 | 5 | 193 | 100 | 164 | 100 |
| 5 | Elongation factor 2 | EF2 | 95,277 | 6.4 | 25 | 565 | 100 | 442 | 100 |
| 6 | Vimentin | VIME | 53,619 | 5.1 | 36 | 1,420 | 100 | 1035 | 100 |
| 7 | Vimentin | VIME | 53,619 | 5.1 | 34 | 1,280 | 100 | 944 | 100 |
| 8 | Trifunctional enzyme subunit alpha, mitochondrial | ECHA | 82,947 | 9.2 | 16 | 302 | 100 | 240 | 100 |
| 9 | Procollagen galactosyltransferase 1 | GT251 | 71,591 | 6.9 | 14 | 283 | 100 | 230 | 100 |
| 10 | Keratin, type II cytoskeletal 6A | K2C6A | 60,008 | 8.1 | 16 | 284 | 100 | 204 | 100 |
| 11 | Keratin, type II cytoskeletal 6B | K2C6B | 60,030 | 8.1 | 11 | 94 | 100 | 53 | 100 |
| 12 | Keratin, type II cytoskeletal 7 | K2C7 | 51,354 | 5.4 | 29 | 849 | 100 | 603 | 100 |
| 13 | Keratin, type I cytoskeletal 17 | K1C17 | 48,076 | 5.0 | 29 | 798 | 100 | 534 | 100 |
| 14 | Keratin, type I cytoskeletal 14 | K1C14 | 51,529 | 5.1 | 26 | 650 | 100 | 457 | 100 |
| 15 | Histone H2B type 1-M | H2B1M | 13,981 | 10.3 | 9 | 309 | 100 | 251 | 100 |

Table legend: MW: molecular weight; PI: isoelectric point; C.I.: confident interval.

**Figure S1**. **Graphical representation of measurements for energy metabolism.** (**A**) Schematic depiction of how basal and maximum respiration values are calculated (**B**) schematic display to show the calculation of glycolysis and maximum glycolysis.

**Figure S2. Glucose uptake kinetics of LNCAP cells.** Time course of (0-30min) of glucose analog uptake kinetics is shown with and without plasma incubation. Data presented are from one single flow cytometry measurement per experimental condition.

**Figure S3. Glucose uptake kinetics of RWPE-1 cells.** Time course of (0-30min) of glucose analog uptake kinetics is shown with and without plasma incubation. Data presented are from one single flow cytometry measurement per experimental condition.

**Figure S4. Lipid uptake kinetics of LNCAP cells.** Time course of (0-30min) of DiD uptake kinetics is shown with and without plasma incubation. Data presented are from one single flow cytometry measurement per experimental condition.

**Figure S5. Lipid uptake kinetics of RWPE-1 cells.** Time course of (0-30min) of DiD uptake kinetics is shown with and without plasma incubation. Data presented are from one single flow cytometry measurement per experimental condition.

**Figure S6. Uptake kinetics of PBMCs for glucose (A) and lipids (B).** Time courses of (0-30min) for fluorescent glucose analog and lipophilic DiD dye uptake kinetics are shown. Data presented are from one single flow cytometry measurement per experimental condition.

**Figure S7. Lipid droplet accumulation in RWPE1 and LNCaP cells following plasma incubation**. RWPE1 and LNCaP cells were incubated for 12 hours with 50% plasma, then fixed, permeabilized and stained with Oil Red O.
